# MicroRNAs in acute kidney injury

**DOI:** 10.1186/s40246-016-0085-z

**Published:** 2016-09-08

**Authors:** Pei-Chun Fan, Chia-Chun Chen, Yung-Chang Chen, Yu-Sun Chang, Pao-Hsien Chu

**Affiliations:** 1 Kidney Research Center, Department of Nephrology, Chang Gung Memorial Hospital, Linkou Medical Center, Taoyuan, Taiwan; 2 Graduate Institute of Clinical Medical Sciences, Chang Gung University, Taoyuan, Taiwan; 3 Molecular Medicine Research Center, Chang Gung University, Taoyuan, Taiwan; 4 Division of Cardiology, Department of Internal Medicine, Chang Gung Memorial Hospital, College of Medicine, Chang Gung University, Taipei, Taiwan; 5 Healthcare Center, Chang Gung Memorial Hospital, College of Medicine, Chang Gung University, Taipei, Taiwan; 6 Heart Failure Center, Chang Gung Memorial Hospital, College of Medicine, Chang Gung University, Taipei, Taiwan; 7 Department of Cardiology, Chang Gung Memorial Hospital, College of Medicine, Chang Gung University, 199 Tung Hwa North Road, Taipei, 105 Taiwan

**Keywords:** MicroRNAs, Acute kidney injury, Renal fibrosis

## Abstract

Acute kidney injury (AKI) is an important clinical issue that is associated with significant morbidity and mortality. Despite research advances over the past decades, the complex pathophysiology of AKI is not fully understood. The regulatory mechanisms underlying post-AKI repair and fibrosis have not been clarified either. Furthermore, there is no definitively effective treatment for AKI. MicroRNAs (miRNAs) are endogenous single-stranded noncoding RNAs of 19~23 nucleotides that have been shown to be crucial to the post-transcriptional regulation of various cellular biological functions, including proliferation, differentiation, metabolism, and apoptosis. In addition to being fundamental to normal development and physiology, miRNAs also play important roles in various human diseases. In AKI, some miRNAs appear to act pathogenically by promoting inflammation, apoptosis, and fibrosis, while others may act protectively by exerting anti-inflammatory, anti-apoptotic, anti-fibrotic, and pro-angiogenic effects. Thus, miRNAs have not only emerged as novel biomarkers for AKI; they also hold promise to be potential therapeutic targets.

## Background

### Acute kidney injury

Acute kidney injury (AKI) is a complex syndrome that occurs in a variety of settings with clinical manifestations ranging from a minimal elevation in serum creatinine to anuric renal failure. AKI conveys significant morbidity and mortality, is a major risk factor of chronic kidney disease, and is thus associated with huge health and socioeconomic burdens [1, 2]. Despite research advances in the past decades, however, the complex pathophysiology of AKI is not fully understood. The regulatory mechanisms underlying post-AKI repair and fibrosis remain to be clarified. Furthermore, there is no definitively effective treatment for AKI.

#### MicroRNA biogenesis and function

MicroRNAs (miRNAs) are endogenous single-stranded noncoding mRNAs of 19~23 nucleotides. They were first discovered in *Caenorhabditis elegans* by Ambros’s group in 1993 [3] and show surprisingly high conservation across species. The evidence accumulated over the past two decades shows that miRNAs play a critical role in the post-transcriptional regulation of almost all biological cell functions, including proliferation, differentiation, metabolism, and apoptosis [4]. miRNAs, which are expressed in a tissue-specific manner, are fundamental to normal development and physiology [4] and are involved in the pathologic pathways of many disease models.

To date, more than 2000 miRNAs have been discovered in the human genome. The miRNA-encoded genes are found as either independent genes having their own promoters, or as sequences in the introns of protein-coding genes [5]. RNA polymerase II transcribes an miRNA gene into a primary transcript (called a pri-miRNA) of several kilobases that can encode either an individual miRNA or a polycistronic cluster of two or more miRNAs. The RNase III enzyme, DROSHA, and its cofactor DGCR8 (Di-George syndrome critical region gene 8 or Pasha), cleave a pri-miRNA at its stem-loop structure, generating an approximately 70-nucleotide intermediate called the pre-miRNA. Exportin-5 exports the pre-miRNA from the nucleus to the cytoplasm, and the RNase III enzyme, DICER, further cleaves it to yield a single-stranded mature miRNA. To perform its function, an miRNA is incorporated along with the argonaute (AGO) protein to form an effector complex called the RNA-induced silencing complex (RISC). RISC binds to the 3′-untranslated region (UTR) of a target messenger RNA (mRNA), leading to the repression of either protein translation or mRNA degradation. Unlike small interfering RNAs in plants, miRNAs do not require complete complementarity to bind their targets. Instead, the evidence suggests that the “seed sequence” (nucleotides 2 through 8 of the miRNA) is the most important region for the ability of an miRNA to bind and regulate its target gene(s). Once bound, miRNAs induce repression by blocking the initiation or elongation of translation or de-adenylating the mRNA transcripts. Because miRNAs do not require complete complementarity to repress gene expression, a given miRNA can regulate multiple mRNA transcripts and a given mRNA transcript can be repressed by multiple miRNAs. It is estimated that miRNAs regulate more than half of the protein-coding genes in human [6]. Moreover, miRNAs have been implicated in various human diseases [7, 8], including kidney diseases, such as polycystic kidney disease [9], renal cell carcinoma [10], diabetic nephropathy [11], lupus nephritis, [12] and renal allograft rejection [13]. In the past few years, researchers have begun to address the relevance of miRNAs to AKI.

#### miRNAs in acute kidney injury

The miRNAs that have been implicated in AKI are summarized in Tables 1 and 2, and those with potential pathological or protective roles are summarized in Table 3. The first evidence of miRNAs having pathological roles in AKI was reported by Wei et al. who developed a *Dicer*-knockout mouse model, in which *Dicer* was specifically deleted from proximal tubular cells. These mice exhibit a global down-regulation of microRNAs in the renal cortex. They have normal renal function and histology under control conditions but show resistance to the AKI that follows bilateral renal ischemia-reperfusion (IRI). Under the latter conditions, *Dicer*-null mice show significantly better renal function, less tissue damage, less tubular apoptosis, and better survival than their wild-type counterparts [14].

**Table 1:** miRNAs implicated in acute kidney injury

miRNA	Samples	Species	Model	Expression	Reference
k12-3	In vitro	HK-2 cells	Oxidative stress	Down then up	[51]
let-7a	T, B	Rat, human	Contrast nephropathy, contrast nephropathy^a^	Down	[52]
let-7a-1-3p	T, U	Rat	Cisplatin nephropathy	Up (urine), down (tissue)	[33]
let-7a-2*	In vitro	HK-2 cells, primary PTCs	AA nephropathy	Up	[38]
let-7b	B	Human	ICU AKI^a^	Down	[46]
let-7d	U	Rat	Gentamicin nephropathy	Down	[53]
let-7e	T, in vitro	Mouse, HK-2 cells	IRI	Up, down	[23, 54]
let-7f	B, T	Human, mouse	ICU AKI^a^, IRI	Down	[34, 46]
let-7g	T, U	Mouse, rat	Cisplatin nephropathy	Up, down	[33, 35]
miR-7	T, in vitro	Mouse, HK-2 cells	IRI, oxidative stress	Up	[14, 51]
miR-7a-1-3p	T, U	Rat	Cisplatin nephropathy	Up (urine), down (tissue)	[33]
miR-10a	T, U, B	Mouse, human, rat	IRI, DM-CKD (STZ), FSGS^a^, ICU AKI^a^	Up, down	[15, 16, 18]
miR-10b*	T	Mouse	Cisplatin nephropathy	Down	[35]
miR-15	U	Rat	Cisplatin nephropathy	Up	[55]
miR-15b-5p	T, in vitro	Mouse, HK-2 cells	IRI	Down	[54]
miR-16	B, U	Human, rat	ICU AKI^a^, cisplatin nephropathy	Up, down	[46, 55]
miR-17-3p	T	Mouse	IRI	Up	[14, 49]
miR-17-5p	T, U	Mouse, rat	IRI, cisplatin nephropathy	Up, down	[19, 20, 33]
miR-18a	T, B, U, in vitro	Mouse, rat, human, HPTECs	IRI, gentamicin nephropathy, folic acid, CdCl_2_, arsenic trioxide, AA, K_2_Cr_2_O_7_, cisplatin, UUO, allograft rejection^a^, renal fibrosis^a^	Up, down	[14, 34, 47, 56, 57]
miR-19a	T	Mouse	IRI	Up	[34]
miR-20a	T, in vitro, U	Mouse, TECs, rat, HK-2 cells	Cisplatin nephropathy, IRI	Up, down	[37, 54, 55]
miR-20b-5p	T, U, in vitro	Rat, mouse, HK-2 cells	Cisplatin nephropathy, IRI	Up (urine), down (tissue)	[33, 54]
miR-21	B, U, T, in vitro	Human, rat, mouse, TEC, CRL-2753 cells, NRK52E cells, HK-2 cells	IRI, TGF-β, anti-Thy 1.1, UUO, SHRSP, gentamicin nephropathy, folic acid, CdCl_2_, arsenic trioxide, AA, K_2_Cr_2_O_7_, allograft rejection^a^, renal fibrosis^a^, AKI^a^	Up, down	[19–30, 34, 37, 45, 56–58]
miR-24	B, T, in vitro	Human, rat, CRL-2753 cells, NRK52E cells, HK-2 cells, HUVECs	ICU AKI^a^, transplantation^a^, UUO, IRI	Up, down	[31, 45, 46]
miR-24-2	T	Mouse	IRI	Up	[34]
miR-25-3p	T, U	Rat	Cisplatin nephropathy	Up (urine), down (tissue)	[33]
miR-26a	In vitro, T	HK-2 cells, mouse	IRI, oxidative stress, cisplatin nephropathy	Down	[32, 35, 51]
miR-26b	T, in vitro, U, B	Rat, CRL-2753 cells, NRK52E cells, human	UUO, cisplatin nephropathy, ICU AKI^a^	Down (tissue, blood), up (urine)	[18, 33, 45]
miR-27a-3p	B	Human	ICU AKI^a^	Down	[18]
miR-29a	T, in vitro, B	HK-2 cells, human	Oxidative stress, ICU AKI^a^	Up, down	[18, 51, 59]
miR-29b	T, in vitro	Rat, HK-2 cells	Oxidative stress	Up	[51, 59]
miR-29c	T	Mouse	IRI	Up	[34, 59]
miR-30a-5p	T, U, in vitro, B	Rat, mouse, HK-2 cells, human	Cisplatin nephropathy, IRI, contrast-induced nephropathy, contrast-induced nephropathy^a^	Up (urine, blood, tissue), down (tissue)	[33, 52, 54]
miR-30c	T, in vitro, B	Rat, CRL-2753 cells, NRK52E cells, mouse, human	TGF-β, UUO, SHRSP, contrast-induced nephropathy, contrast-induced nephropathy^a^	Up, down	[34, 45, 52]
miR-30c-1	T	Mouse	IRI	Up	[34]
miR-30c-2*	In vitro	HK-2 cells	Oxidative stress	Down	[51]
miR-30d	T, U, B	Mouse, human	IRI, DM-CKD (STZ), FSGS^a^	Up, down, unchanged	[16]
miR-30d*	B	Human	ICU AKI^a^	Down	[46]
miR-30e	T, B	Mouse, rat, human	Cisplatin nephropathy, contrast-induced nephropathy, contrast-induced nephropathy^a^	Up, down	[35, 52]
miR-34a	T, in vitro	Mouse, BUMPT-306 cells, NRK-52E cells, RTECs	Cisplatin nephropathy, IRI	Up	[35, 60, 61]
miR-34b	T	Mouse	IRI	Up	[47]
miR-92a	T	Mouse	IRI	Up	[34]
miR-92b*	B	Human	ICU AKI^a^	Up	[46]
miR-93-3p	B	Human	ICU AKI^a^, AKI post-cardiac surgery^a^	Down	[18]
miR-93-5p	T, U	Rat	Cisplatin nephropathy	Up (urine), down (tissue)	[33]
miR-99b	In vitro, T	HK-2 cells, mouse	ER stress, IRI	Down	[51, 54]
miR-101-3p	B	Human	ICU AKI^a^	Down	[18]
miR-101a	T, in vitro	Mouse, HK-2 cells	UUO	Down	[25]
miR-106a-5p	T, in vitro	Mouse, HK-2 cells, primary PTCs, rat	IRI, AA nephropathy	Up, down	[19, 20, 38]
miR-122	T	Mouse	Cisplatin nephropathy, IRI	Down	[35, 49]
miR-123	T	Mouse	IRI	Up	[49]
miR-125a-5p	T, in vitro	Mouse, HK-2 cells	IRI	Down	[54]
miR-125b	T, in vitro	Mouse, HepG2 cells, HEK293 cells, NRK52E cells	Cisplatin nephropathy		[62]
miR-126-3p	B	Human	ICU AKI^a^	Down	[18]
miR-126-5p	T, in vitro	Mouse, rat, TEnCs, TEpCs	IRI	Up	[34, 43, 44, 63]
miR-127-3p	T, in vitro, B	Rat, mouse, NRK-52E cells, HK-2 cells, human	IRI, ICU AKI^a^, AKI post-cardiac surgery^a^	Up, down	[14, 18, 36, 49]
miR-129-3p	T	Mouse	IRI	Up	[34]
miR-129-5p	In vitro	HK-2 cells, primary PTCs	AA nephropathy	Down	[38]
miR-130b-3p	T, U	Rat	Cisplatin nephropathy	Up (urine), down (tissue)	[33]
miR-132	T, in vitro	Mouse, human, HPTECs	IRI, folic acid, CdCl_2_, arsenic trioxide, AA, K_2_Cr_2_O_7_, cisplatin, UUO, allograft rejection^a^, renal fibrosis^a^	Up	[14, 57]
miR-133a	In vitro	HK-2 cells	ER stress	Down	[51]
miR-134	T	Mouse	IRI	Up	[47]
miR-135b	T	Mouse	IRI	Down	[14, 49]
miR-140-3p	T, U	Rat	Cisplatin nephropathy	Up (urine), down (tissue)	[33]
miR-141	T	Mouse	IRI	Up	[34]
miR-142-3p	T, in vitro	Mouse, HK-2 cells	UUO	Up	[25]
miR-142-5p	T, in vitro	Mouse, HK-2 cells	UUO	Up	[25]
miR-145	T, in vitro	Rat, mouse, CRL-2753 cells, NRK52E cells, CD133+ renal medullary progenitor cells	TGF-β, SHRSP salt challenge	Up, down	[39, 45, 64]
miR-146a	T, in vitro, B	Mouse, TECs, human	IRI, ICU AKI^a^	Down (blood), up (tissue)	[18, 37]
miR-146b-5p	T, in vitro	Mouse, human, HPTECs	IRI, folic acid, CdCl_2_, arsenic trioxide, AA, K_2_Cr_2_O_7_, cisplatin, UUO, allograft rejection^a^, renal fibrosis^a^	Up	[57]
miR-149	T	Mouse	IRI	Down	[34]
miR-150	T, in vitro	Mouse, immortalized mouse cardiac endothelial cell lines	IRI, AMI using LAD ligation	Down	[65]
miR-155	B, U, T, in vitro	Rat, human, mouse, HK-2 cells	IRI, gentamicin nephropathy, Cisplatin nephropathy, AKI^a^	Up, down	[54, 56, 66]
miR-181a*	In vitro	HK-2 cells	ER stress	Up	[51]
miR-181a-2*	In vitro	HK-2 cells	ER stress	Down	[51]
miR-181d	T	Mouse	IRI	Down	[34]
miR-182	T	Mouse	IRI	Up	[47]
miR-183-5p	T, U	Rat	Cisplatin nephropathy	Up (urine), down (tissue)	[33]
miR-187	T, in vitro	Mouse, TECs	IRI	Down	[37]
miR-188-5p	T	Mouse	IRI	Up	[34]
miR-191a-5p	T, U	Rat	Cisplatin nephropathy	Up (urine), down (tissue)	[33]
miR-192	T, in vitro, B, U	Mouse, rat, CRL-2753 cells, NRK52E cells, TECs, HK-2 cells, primary PTCs	IRI, UUO, SHRSP, AA nephropathy, cisplatin nephropathy, contact freezing, Dahl salt-sensitive rat with high salt administration	Up, down	[15, 33, 45, 55]
miR-193	T, in vitro, U	Mouse, HK-2 cells, Rat	UUO, cisplatin nephropathy	Down (tissue), up (urine)	[25, 33, 35, 55]
miR-194	T, in vitro, B, U	Mouse, rat, TECs, HK-2 cells, primary PTCs	IRI, AA nephropathy, contact freezing, Dahl salt-sensitive rat with high salt administration	Up, down	[15, 37–39]
miR-197	T	Mouse	IRI	Down	[34]
miR-199a-3p	T, in vitro	Mouse, TECs	IRI	Up	[37]
miR-200a	T, B, U	Human, Rat, mouse	Contact freezing, Dahl salt-sensitive rat with high salt administration, contrast-induced nephropathy, contrast-induced nephropathy^a^	Up, down	[39, 52]
miR-200b	T, in vitro, B, U	Rat, CRL-2753 cells, NRK52E cells, human	TGF-β, UUO, contact freezing, early CKD (Dahl salt-sensitive rat with high salt administration)	Up, down	[34, 39, 45]
miR-200c	T, in vitro, U, B	Rat, CRL-2753 cells, NRK52E cells, human	TGF-β, contact freezing, early CKD (Dahl salt-sensitive rat with high salt administration), ICU and transplant AKI^a^	Up, down	[29, 39, 45]
miR-202	In vitro	HK-2 cells	ER stress	Down	[51]
miR-203	U	Rat	Gentamicin nephropathy	Down	[53]
miR-205	In vitro	HK-2 cells, primary PTCs	Oxidative stress, ER stress, AA nephropathy	Down	[38, 51]
miR-207	T	Mouse	IRI	Up, down	[14, 34]
miR-210	B, T, in vitro, U	Human, mouse, HUVEC-12 cells, HK-2 cells, primary PTCs, rat	IRI, Oxidative stress, AA nephropathy, cisplatin nephropathy, ICU AKI^a^	Up, down	[18, 34, 38, 46, 47, 51, 55]
miR-211	T	Mouse	IRI	Down	[34]
miR-212	T	Mouse, human, HPTECs	IRI, folic acid, CdCl_2_, arsenic trioxide, AA, K_2_Cr_2_O_7_, cisplatin, UUO, allograft rejection^a^, renal fibrosis^a^	Up, down	[34, 57]
miR-214	T, in vitro	Mouse, rat, HK-2 cells, TECs, CRL-2753 cells, NRK52E cells, human	TGF-β, anti-Thy 1.1, UUO, SHRSP, IRI, diabetic nephropathy^a^	Up	[23–25, 37, 45, 47]
miR-215	In vitro	HK-2 cell	ER stress	Down	[51]
miR-218	T, in vitro	Mouse, HK-2 cells	UUO	Down	[25]
miR-218-1	T	Mouse	IRI	Up	[34]
miR-218a-5p	T, U	Rat	Cisplatin nephropathy	Up (urine),down (tissue)	[33]
miR-221*	In vitro	HK-2 cells	Oxidative stress	Up	[51]
miR-223	T, in vitro	Mouse, HK-2 cells	UUO	Up	[25]
miR-290-3p	T	Mouse	IRI	Up	[34]
miR-296	T, in vitro	Rat, mouse, TEnCs, TEpCs	IRI	Up, down	[14, 43]
miR-302b	T	Mouse	IRI	Up	[34]
miR-302c	T	Mouse	IRI	Up	[34]
miR-320	B, T, U	Human, mouse, rat	IRI, cisplatin nephropathy, gentamicin nephropathy, contrast-induced nephropathy, ICU AKI^a^, contrast-induced nephropathy^a^	Up, down	[23, 33, 34, 46, 52, 53]
miR-322	T	Mouse	IRI	Down	[14]
miR-324-3p	T	Mouse	IRI	Down	[14]
miR-326	T	Mouse	IRI	Down	[34]
miR-328	T	Mouse	IRI	Down	[34]
miR-328a-3p	T, U	Rat	Cisplatin nephropathy	Up (urine),down (tissue)	[33]
miR-329	T, in vitro	Rat, CRL-2753 cells, NRK52E cells	UUO	Down	[45]
miR-335	T, U	Rat	Cisplatin nephropathy	Up (urine),down (tissue)	[33]
miR-340-5p	T, U	Rat	Cisplatin nephropathy	Up (urine),down (tissue)	[33]
miR-346	T	Mouse	IRI	Down	[34]
miR-362-5p	T	Mouse	IRI	Up	[14, 34]
miR-365*	In vitro	HK-2 cells, primary PTCs	AA nephropathy	Down	[38]
miR-378a-5p	T, U	Rat	Cisplatin nephropathy	Up (urine),down (tissue)	[33]
miR-379	T	Mouse	IRI	Down	[14, 49]
miR-382	In vitro	HK-2 cells, primary PTCs	AA nephropathy	Up	[38]
miR-423	U	Human	ICU and transplant AKI^a^	Up	[29]
miR-449	In vitro	NRK-52E cells	Cisplatin nephropathy	Up	[67]
miR-450a-3p	T, in vitro	Mouse, HK-2 cells, primary PTCs	IRI, AA nephropathy	Up, down	[34, 38]
miR-451	T	Mouse	IRI	Up	[34]
miR-455-3p	T	Mouse	IRI	Down	[14]
miR-466a-5p	T	Mouse	IRI	Up	[34]
miR-466b-5p	T	Mouse	IRI	Up	[34]
miR-466c-5p	T	Mouse	IRI	Down	[34]
miR-466f-3p	T	Mouse	IRI	Down	[34]
miR-466g	T	Mouse	IRI	Down	[34]
miR-466i	T	Mouse	IRI	Down	[34]
miR-467	T	Mouse	IRI	Up	[14]
miR-467a	T	Mouse	IRI	Down	[34]
miR-467b	T	Mouse	IRI	Down	[34]
miR-467e	T	Mouse	IRI	Down	[34]
miR-467f	T	Mouse	IRI	Down	[34]
miR-467g	T	Mouse	IRI	Down	[34]
miR-468	T	Mouse	IRI	Down	[34]
miR-483	T	Mouse	IRI	Up, down?	[34]
miR-484	T	Mouse	IRI	Down	[34]
miR-486	T	Mouse	IRI	Up	[14]
miR-487b	T	Mouse	IRI	Down	[14]
miR-489	T	Mouse	IRI	Up	[14]
miR-491	T	Mouse	IRI	Down	[14]
miR-494	T, U, B	Mouse, human	IRI, ICU AKI^a^	Up, unchanged	[48]
miR-495	T	Mouse	IRI	Up	[14]
miR-503	In vitro	HK-2 cells	ER stress	Down	[51]
miR-532-3p	T, U	Mouse, rat	IRI, Cisplatin nephropathy	Up, down	[33, 34]
miR-542-3p	In vitro	HK-2 cells, primary PTCs	AA nephropathy	Up	[38]
miR-547-3p	T	Mouse	IRI	Down	[34]
miR-574-5p	In vitro	HK-2 cells, primary PTCs	AA nephropathy	Down	[38]
miR-617	B	Human	ICU AKI^a^	Up	[46]
miR-620	B	Human	ICU AKI^a^	Down	[46]
miR-625*	In vitro	HK-2 cells, primary PTCs	AA nephropathy	Down	[38]
miR-630	In vitro	HK-2 cells	Oxidative stress	Up	[51]
miR-638	B	Human	ICU AKI^a^	Up	[46]
miR-663b	B	Human	ICU AKI^a^	Up	[46]
miR-668	T	Mouse	IRI	Up	[14]
miR-669a	T	Mouse	IRI	Down	[34]
miR-669f	T	Mouse	IRI	Down	[34]
miR-669h-3p	T	Mouse	IRI	Down	[34]
miR-671-3p	In vitro	HK-2 cells, primary PTCs	AA nephropathy	Down	[38]
miR-671-5p	T	Mouse	IRI	Up	[34]
miR-674	T	Mouse	IRI	Down	[34]
miR-680	T	Mouse	IRI	Up	[34]
miR-684	T	Mouse	IRI	Up	[34]
miR-685	T	Mouse	IRI	Up	[14, 34, 49]
miR-687	T, in vitro	Mouse, BUMPT-306 cells, HEK cells	IRI	Up	[14, 49]
miR-689	T	Mouse	IRI	Up	[34]
miR-694	T	Mouse	IRI	Up	[14]
miR-705	T	Mouse	IRI	Up	[34]
miR-708	T	Mouse	IRI	Up	[34]
miR-714	T, B	Mouse	IRI	Up	[68]
miR-718	T	Mouse	IRI	Down	[34]
miR-721	T	Mouse	IRI	Up	[34]
miR-744-5p	T, U	Rat	Cisplatin nephropathy	Up (urine),down (tissue)	[33]
miR-805	T, in vitro	Mouse, TECs	IRI	Down	[34, 37]
miR-875-5p	T	Mouse	IRI	Down	[34]
miR-876-5p	T	Mouse	IRI	Up	[34]
miR-877	T	Mouse	IRI	Up, down?	[34]
miR-877*	T, B	Mouse	IRI	Up	[68]
miR-1187	T	Mouse	IRI	Down	[34]
miR-1188	T, B	Mouse	IRI	Up	[68]
miR-1196	T	Mouse	IRI	Down	[34]
miR-1198	T	Mouse	IRI	Down	[34]
miR-1224	T, B	Mouse	IRI	Up	[68]
miR-1244	B	Human	ICU AKI^a^	Down	[46]
miR-1249	In vitro	HK-2 cells, primary PTCs	AA nephropathy	Up	[38]
miR-1839-5p	T, U	Rat	Cisplatin nephropathy	Up (urine),down (tissue)	[33]
miR-1892	T	Mouse	IRI	Up	[34]
miR-1894-3p	T	Mouse	IRI	Up	[34]
miR-1897-3p	T, B	Mouse	IRI	Up	[68]
miR-4521	In vitro	HK-2 cells, primary PTCs	AA nephropathy	Down	[38]
miR-4640	U	Human	ICU and transplant AKI^a^	Down	[29]
miR-4716-5p	In vitro	HK-2 cells, primary PTCs	AA nephropathy	Up	[38]
miR-4730	In vitro	HK-2 cells, primary PTCs	AA nephropathy	Up	[38]
miR-4747-3p	In vitro	HK-2 cells, primary PTCs	AA nephropathy	Up	[38]

^a^Human studies

**Table 2:** miRNAs implicated in human studies related to kidney injury

miRNA	Kidney injury	Expression		Reference
		Up	Down	
hsa-let-7b	AKI in ICU		Blood	[46]
hsa-let-7f	AKI in ICU		Blood	[46]
hsa-miR-10a	Focal segmental sclerosis	Urine		[16]
	AKI in ICU		Blood	[18]
hsa-miR-16	AKI in ICU		Blood	[46]
hsa-miR-21	AKI, chronic renal allograft dysfunction, renal allograft rejection, renal fibrosis	Tissue, blood, urine		[24, 26, 28, 29, 56]
	AKI after cardiac surgery		Blood	[30]
hsa-miR-24	AKI in ICU		Blood	[46]
	Transplanted renal graft with prolonged cold ischemia time	Tissue		[31]
hsa-miR-26b	AKI in ICU		Blood	[18]
hsa-miR-27a-3p	AKI in ICU		Blood	[18]
hsa-miR-29a	AKI in ICU		Blood	[18]
hsa-miR-30a-5p	Contrast-induced nephropathy	Blood		[52]
hsa-miR-30c	Contrast-induced nephropathy	Blood		[52]
hsa-miR-30d	Focal segmental sclerosis	Urine		[16]
hsa-miR-30d*	AKI in ICU		Blood	[46]
hsa-miR-30e	Contrast-induced nephropathy	Blood		[52]
hsa-miR-92b*	AKI in ICU	Blood		[46]
hsa-miR-93-3p	AKI in ICU, AKI post-cardiac surgery		Blood	[18]
hsa-miR-101-3p	AKI in ICU		Blood	[18]
hsa-miR-126-3p	AKI in ICU		Blood	[18]
hsa-miR-127-3p	AKI in ICU, AKI post-cardiac surgery		Blood	[18]
hsa-miR-146a	AKI in ICU		Blood	[18]
hsa-miR-155	AKI	Urine		[56]
hsa-miR-200c	AKI in ICU, AKI in renal transplant	Urine		[29]
hsa-miR-210	AKI in ICU	Blood		[46]
	AKI in ICU		Blood	[18]
hsa-miR-214	Diabetes related chronic kidney disease stage 4	Tissue		[24]
hsa-miR-320	AKI in ICU		Blood	[46]
hsa-miR-423	AKI in ICU, AKI in renal transplant	Urine		[29]
hsa-miR-494	AKI in ICU	Urine		[48]
hsa-miR-617	AKI in ICU	Blood		[46]
hsa-miR-620	AKI in ICU		Blood	[46]
hsa-miR-638	AKI in ICU	Blood		[46]
hsa-miR-663b	AKI in ICU	Blood		[46]
hsa-miR-1244	AKI in ICU		Blood	[46]
hsa-miR-4640	AKI in ICU, AKI in renal transplant		Urine	[29]

**Table 3:** Functional roles of miRNAs in acute kidney injury

Protective		Pathogenic	Kidney enriched, released from injured kidney tissues
Anti-inflammation miR-10a miR-21 miR-26a miR-126 miR-146a miR-199a miR-296 Anti-apoptosis miR-10a miR-21 miR-122 miR-126 miR-199a miR-296 miR-494 Anti-fibrosis miR-29a miR-200b miR-200c	Pro-angiogenesis miR-126 miR-210 miR-296 Enhancing tubular proliferation miR-126 miR-296 Cytoskeleton, cell-matrix, cell-cell adhesion, cell trafficking miR-127a	Pro-inflammation miR-21 miR-214 miR-494 Pro-apoptosis miR-24 miR-192 miR-494 miR-687 Pro-fibrosis miR-21 miR-192 miR-214	miR-10a miR-30c miR-30d miR-200 family

miR-10a is renal tubule-specific miRNA that is released from kidney tissues upon injury. In rodent models of renal IRI and streptozocin (STZ)-induced diabetic nephropathy, the levels of miR-10a are increased decreased in urine and kidney tissue, respectively [15, 16]. miR-10a is thought to exert protective actions during injury by targeting IL-12/IL-23p40 and the pro-apoptotic protein BIM [17]. In humans, decreased plasma levels of miR-10a have been shown to predict AKI in critical patients of intensive care units (ICUs) [18].

The members of the miR-17 family have been shown to be induced by pro-inflammatory cytokines, and their tissue expressions are increased in rodent models of renal IRI [19, 20].

miR-21 appears to play a dual role; on the one hand, it protects against injury by inhibiting apoptosis and inflammation; on the other hand, it may amplify the injury response and promote fibrosis. Studies have shown that miR-21 inhibits apoptosis by down-regulating programmed cell death protein 4 (PDCD4), down-regulating phosphatase and tensin homolog (PTEN), activating the AKT pathway, up-regulating B cell lymphoma 2 (BCL-2), and decreasing the levels of active caspase-3 and caspase-8 proteins [21, 22]. Up-regulation of miR-21 also inhibits inflammation by decreasing nuclear factor-kappaB (NF-kB), tumor necrosis factor (TNF), interleukin 6 (IL-6), and IL-18, and by increasing IL-10 [21]. Experimental up-regulation of miR-21 provides morphologic and functional renoprotection in animal models of AKI [21–23]. miR-21 is induced by transforming growth factor beta (TGF-β)/Smad, hypoxia inducible factor 1 alpha (HIF-1α), TNF, and fibroblast growth factor 2 (FGF-2) [24, 25], and this miRNA promotes fibrosis by targeting peroxisome proliferator-activated receptor alpha (Pparα) and altering lipid metabolism [26]. miR-21 also targets Mpv17l, a mitochondria inhibitor of reactive oxygen species (ROS) [26]. miR-21 inhibits autophagy by targeting Ras-related proteins in brain 11 a (Rab-11a), decreasing light chain 3-II (LC3-II), decreasing beclin-1, and increasing p62 [27]. In vivo blockade of miR-21 reduces renal fibrosis and macrophage infiltration in animal models. Moreover, increased urinary and plasma levels of miR-21 have been observed in various clinical AKI settings [26, 28, 29]. For example, urine and plasma miR-21 levels were shown to correlate with AKI severity and hospital mortality and to predict the need for postoperative renal replacement therapy [28]. Interestingly, one study found decreased, but not increased, expression of miR-21 in AKI patients. Lower baseline plasma levels of miR-21 have been demonstrated to predict cardiac surgery-associated AKI [30].

miR-24 is up-regulated in mouse kidney after IRI and in patients after kidney transplantation. This miRNA enhances apoptosis by down-regulating sphingosine-1-phosphate receptor 1 (S1PR1), H2A histone family member X (H2A.X), and heme oxygenase-1 (HO-1). Inhibition of miR-24 was shown to prevent renal injury in animal models [31].

miR-26a represses IL-6 expression to promote the expansion of regulator T cells (Tregs). The tissue levels of miR-26a is down-regulated in animal models of AKI, and experimental overexpression attenuates renal IRI and improves renal recovery [32]. miR-26b is down-regulated in the tissue and blood, yet up-regulated in the urine [18, 33]. Decreased blood levels of miR-26a and miR-27a predict AKI in the ICU. Decreased blood levels of miR-26a and miR-27a prior to cardiac surgery also predict AKI later on [18].

miR-29a is highly expressed in the kidney, where it acts against fibrosis by suppressing collagen expression in tubular cells. Decreased serum levels of miR-29a have been shown to predict AKI in ICU patients, and correlate with AKI severity [18].

miR-30c, which is essential for normal kidney homoeostasis, targets several genes important for kidney structure and function. miR-30c is up-regulated in the tissue, blood, and urine obtained from animal models of contrast nephropathy and IRI [34].

miR-30d, which is released to the urine from kidney tissues following injury, down-regulates the apoptotic proteins, caspase 3 and p53, and may provide protective effects during IRI [16].

miR-101-3p is highly expressed in the kidney, and decreased serum levels of this miRNA have been shown to predict AKI in the ICU [18].

miR-122 is down-regulated in the mice kidneys of mice subjected to cisplatin-induced AKI [35]. It exerts anti-apoptotic effects by down-regulating forkhead box O3 (Foxo3).

miR-127a, which is induced by HIF-1α, participates in protecting the cytoskeleton protection (by preventing actin depolmerization), maintaining cell-matrix and cell-cell adhesion maintenance (by preventing focal adhesion complexes disassembly and tight junctions disorganization), and promoting intracellular trafficking (by targeting kinesin family member 3B) [36]. Decreased blood levels of miR-127a were shown to predict AKI in the ICU. Decreased blood levels of miR-127a prior to cardiac surgery were found be predict AKI later on [18].

miR-146a is down- and up-regulated in the blood and kidney, respectively, during AKI. Decreased blood levels have been shown to predict AKI in the ICU and correlate with the severity of AKI [18]. It is induced by NF-kB and exerts anti-inflammatory effect by down-regulating TNF receptor-associated factor 6 (TRAF-6) and interleukin-1 receptor-associated kinase 1 (IRAK-1) [37].

miR-192 is enriched in kidneys and the small intestine. It is induced by TGF-β during the stress response. It promotes fibrosis by down-regulating SIP1. It also down-regulates E3 ubiquitin ligase and murine double-minute 2 (MDM2) and results in de-repression of p53 and G2/M arrest [38]. miR-194 is also enriched in kidneys and small intestine. It is induced during the stress response, and its levels in tissue, blood, and urine levels are increased during AKI [15, 38, 39].

miR-199a exerts anti-inflammatory effect by down-regulating inhibitor of NF-kB kinases b (IKKb) [40], exhibits anti-proliferatory effect by down-regulating the proto-oncogene MET [41], and confers anti-apoptosis effect by down-regulating extracellular signal–regulated kinase 2 (ERK-2) and HIF-1α [41, 42]. Therefore, it may help limit kidney injury.

miR-126 and miR-296 have been identified in microvesicles from endothelial progenitor cells and are thought to exert renoprotective effects via their abilities to decrease apoptosis and leukocyte infiltration, while promotes angiogenesis and tubular cell proliferation [43]. Hematopoietic overexpression of miR-126 enhances stromal cell-derived factor 1/chemokine receptor type 4 (CXCR4) -dependent vasculogenic progenitor cell mobilization and promotes vascular integrity and supports renal recovery after IRI [44]. Decreased serum levels of miR-126 have been shown to predict AKI in ICU patients, and correlate with the severity of AKI [18].

Members of the miR-200 family are highly expressed in tubular structures such as renal tubules, lungs, the small intestine, and various exocrine glands. miR-200b and miR-200c have been proposed to be anti-fibrotic. They down-regulate TGFβR1 and zinc finger E-box-binding homeobox (ZEB1/ZEB2), which are transcriptional repressors of E-cadherin, and thereby prevent the epithelial-to-mesenchymal transition (EMT) induced by TGF-β [45].

miR-210 is induced by HIF1-α and released by renal endothelial cell. It regulates angiogenesis by down-regulating ephrin-A3 and up-regulating vascular endothelial growth factor (VEGF) and vascular endothelial growth factor receptor 2 (VEGFR2). It also regulates mitochondria ROS production. Increased blood levels of miR-210 was shown to predict post-AKI mortality in critically ill patients [46]. In another study, decreased blood levels of miR-210 were shown to predict AKI in the ICU and correlate with the severity of AKI [18].

miR-214 is induced by TGF-β and promotes fibrosis; it has been shown to down-regulate PTEN, up-regulate the AKT pathway and inhibit apoptosis of monocytes and macrophages. miR-214 is up-regulated in various models of AKI and renal fibrosis [24, 45, 47] as well as in monocytes of animal with chronic kidney disease. Experimental antagonism of miR-214 has been shown to ameliorate renal fibrosis [24].

miR-494 is up-regulated early in AKI, with increased urine levels detected in rodent models of renal IRI and patients with AKI. It has been reported to promote apoptosis and inflammation by down-regulating activating transcription factor 3 (ATF3) and increasing IL-6, monocyte chemoattractant protein-1 (MCP-1), p-selectin [48]. Pathway analysis has suggested that it also targets adiponectin receptor 2 (ADIPOR2), BCL-2 facilitator, and insulin-like growth factor 1 receptor (IGF1R), which would increase inflammation and lead to more damage. However, miR-494 also targets pro-apoptotic proteins in the AKT pathway, and to exert protective effects. The mechanism responsible for regulating the balance between these anti- and pro- apoptotic effects requires further study.

Finally, miR-687 is induced by HIF-1, and enhances apoptosis by down-regulating PTEN. Animal studies have shown that miR-687 blockade preserves PTEN expression and attenuates cell cycle activation and decreases apoptosis, resulting in protection against kidney injury [49].

## Conclusions

Many miRNAs have been implicated in the AKI. Some of them contribute to the pathogenesis by regulating apoptosis and inflammation, to amplifying or reduce acute injury responses, while others regulate fibrosis and angiogenesis, to participate in renal recovery or the progression to fibrosis. The biological and pathological functions of many miRNAs in AKI are still not fully understood in AKI. Some studies have yielded inconsistent data regarding the expression pattern of miRNAs across different samples, species, disease models, and time points. These discrepancies warrant investigations.

In addition to their tissue expressions, miRNAs may be detected in various extracellular human body fluids, such as serum, urine, saliva, and cerebral spinal fluid. miRNAs are contained in exosomes and may remained stable over prolonged periods. They may be specifically up-regulated or down-regulated in response to injury signals and/or released into body fluids from resident tissues. Certain miRNAs have been investigated for their potential to serve as novel biomarkers for the early detection or prognostication of AKI. Given the complex pathophysiology and the dynamic nature of AKI, an miRNA panel may be more feasible rather than a single miRNA. Further validation studies are needed to evaluate the clinical utility of such a panel.

Some miRNAs may be potential therapeutic targets for AKI. Recently, an miRNA inhibitor has been proven to successfully suppress the replication of hepatitis C virus in a clinical trial [50]. Systemic or local administration of specific miRNAs mimics or antagonists in vivo could offer a strategy for preventing or ameliorating AKI or barring its progression to chronic kidney disease.

In the post-genome era, miRNAs are promising rising stars in translational medicine as they offer the potential to guide the individualized diagnosis and treatment of human diseases including AKI.

## Abbreviations

AA: aristolochic acid
ADIPOR2: adiponectin receptor 2
AGO: argonaute
AKI: acute kidney injury
ATF3: activating transcription factor 3
B: blood
BCL-2: B cell lymphoma 2
BUMPT-306 cell: Boston University mouse proximal tubule cell clone 306
CdCl_2_: cadmium chloride
CRL-2753 cell: rat mesangial cell line
CKD: chronic kidney disease
CXCR4: chemokine receptor type 4
DGCR8: Di-George syndrome critical region gene 8 or Pasha
DM: diabetes mellitus
EMT: epithelial-to-mesenchymal transition
ER: endoplasmic reticulum
ERK-2: extracellular signal-regulated kinase 2
FGF-2: fibroblast growth factor 2
Foxo3: forkhead box O3
FSGS: focal segmental glomerulosclerosis
H2A.X: H2A histone family member X
HEK cell: human embryonic kidney cell
HepG2 cell: human hepatocellular liver carcinoma cell line
HIF-1α: hypoxia-inducible factor 1 alpha, HK-2 cell, human kidney 2 cell
HO-1: heme oxygenase-1
HPTEC: human proximal tubular epithelial cell
HUVEC: human umbilical vein endothelial cell
ICU: intensive care units
IGF1R: insulin-like growth factor 1 receptor
IL: interleukin
IKKb: inhibitor of NF-kB kinases b
IRAK-1: interleukin-1 receptor-associated kinase 1
IRI: ischemia-reperfusion injury
K_2_Cr_2_O_7_: potassium dichromate
LC3-II: light chain 3-II
MCP-1: monocyte chemoattractant protein-1
MDM2: murine double-minute 2
miRNA: microRNA
mRNA: messenger RNA
NF-kB: nuclear factor-kappaB
NRK-52E cell: rat renal proximal tubular cell line
PDCD4: programmed cell death protein 4
Pparα: peroxisome proliferator activated receptor alpha
PTC: proximal tubular cell
PTEN: phosphatase and tensin homolog
Rab-11a: Ras-related proteins in brain 11 a
RISC: RNA-induced silencing complex
ROS: reactive oxygen species
S1PR1: sphingosine-1-phosphate receptor 1
SHRSP: stroke-prone spontaneously hypertensive rat
STZ: streptozocin
T: tissue
TEC: tubular epithelial cell
TEnC: tubular endothelial cell
TEpC: tubular epithelial cell
TNF: tumor necrosis factor
TGF-β: transforming growth factor beta
TRAF-6: TNF receptor-associated factor 6
Treg: regulator T cell
U: urine
UTR: untranslated region
UUO: unilateral ureteral obstruction
VEGF: vascular endothelial growth factor
VEGFR2: vascular endothelial growth factor receptor 2
ZEB1/ZEB2: zinc finger E-box-binding homeobox
